# Changing bulking agent may require change in injection volume for endoscopic treatment of vesicoureteral reflux

**DOI:** 10.1590/S1677-5538.IBJU.2018.0033

**Published:** 2018

**Authors:** Ali Tekin, Ismail Yagmur, Sibel Tiryaki, Zafer Dokumcu, Ibrahim Ulman, Ali Avanoglu

**Affiliations:** 1 Department of Pediatric Surgery, Ege University Faculty of Medicine, Izmir, Turkey;; 2 Division of Pediatric Urology, Ege University Faculty of Medicine, Izmir, Turkey

**Keywords:** Vesico-Ureteral Reflux, Endoscopy, Cakut [Supplementary Concept]

## Abstract

**Introduction:**

Various bulking agents were utilized for endoscopic correction of VUR. A study reviewing multi-institutional data showed that the amount of injection material has increased over time with the purpose of improving success rates, which also resulted in costs. We noticed an opposite trend in our center since we started using a new bulking agent. The aim of this study was to evaluate evolution of our practice with different bulking agents.

**Patients and Methods:**

Records of VUR patients who underwent subureteric injection with polyacrylate polyalcohol copolymer (PPC) and dextronomere hyaluronic acide (DxHA) between 2005 and 2014 were reviewed. Variation of different parameters throughout the study period was evaluated along with the success rate. Success was defined as complete resolution of reflux.

**Results:**

A total of 260 patients with 384 refluxing units were included. The success rate was higher in PPC group compared to DxHA group. There was no statistically significant difference between years regarding distribution of VUR grade, body weight, patient height, and age in PPC group. Despite significant reduction in injection volume, success rate did not decrease through the years with PPC.

**Conclusion:**

Different bulking agents may require different injection volumes to achieve the same success rate in endoscopic treatment of vesicoureteral reflux. Habits gained with previous experience using other materials should be revised while using a new agent.

## INTRODUCTION

Endoscopic subureteric injection has become the most popular surgical method in the management of vesicoureteral reflux (VUR) in children, largely due to lower complication rates and the ease of application (1-4). Essential features of an ideal bulking agent are: easy applicability, inducing less tissue reaction, volume - stability, non - antigenicity, and being non - migratory (4). In recent years, different bulking agents were used for endoscopic correction of VUR and some of them became very popular (3-6). A study reviewing multi - institutional data showed that the amount of injection material has increased over time to improve success rates, although they resulted in higher treatment costs (7). We noticed an opposite trend in our center, since we started using a new bulking agent. The aim of this study was to evaluate progression of our practice with different bulking agents.

## PATIENTS AND METHODS

We reviewed the hospital records of VUR patients who had undergone subureteric injection with dextranomer hyaluronic acid (DxHA) and polyacrilate polyalcohol copolymer (PPC) in our institution between 2005 and 2014. Data including patient demographics, injected material volumes, VUR types (primary or secondary), and VUR grades according to the pre - and postoperative voiding cystourethrograms (VCUG) and success rates were similarly recorded. Variation of different parameters throughout the study period was evaluated along with the success rate. Reflux was classified according to the International Reflux Study Committee's Classification System. The procedure was performed under general anesthesia using 8 Fr 6° cystoscope (Storz^®^, Tutlingen, Germany). Subureteric injection either with Polyacrilate polyalcohol copolymer (PPC) (Vantris^®^, Promedon, Argentina) or DxHA (Dexell İstem Medikal, Turkey) was administered slowly using a Williams cystoscopic injection needle (Cook Medical, Bloomington, USA) submucosally at the 6 o'clock position of the ureteral orifice until creating a prominent bulge. Evaluation and management of bladder dysfunction was completed before the injection procedure in secondary reflux cases. Success was defined as complete resolution of reflux in VCUG obtained at least three months after the injection. Injection was repeated if persistent reflux above grade 1 was documented. Ultrasonography was performed at the postoperative first, third, and sixth months, and then annually for follow-up of obstructive findings like new onset or increasing hydronephrosis. Statistical analysis was carried out with the SPSS statistical package (SPSS for windows V.16, SPSS, Chicago, IL, USA) and Pearson Chi - square, Mann - Whitney U, Kruskal Wallis tests as required.

## RESULTS

A total of 260 patients including 71 patients with 101 refluxing units in DxHA group and 189 patients with 283 refluxing units in PPC group were included in the study. VUR was primary in 73.3% and secondary (bladder - sphincter dysfunction) in 26.7% in DxHA group and primary in 79.9% and secondary in 20.1% in PPC group. Number of patients, mean ages, number of refluxing units, mean injected volumes and success rates are summarized in Table-1. There was no statistically significant difference between groups regarding reflux type, gender, and reflux grade. However, mean injected volume was significantly lower in PPC group (p < 0.05). The success rate was higher in PPC group compared to DxHA group (p < 0.05, Mann Whitney U test).

**Table 1:** Comparison of PPC and DxHA groups.

	PPC	DxHA	p
Number of Patients (G/B) *	189 (111/78)	71 (44/27)	
Mean Age (Years)	4.8 ± 3.8	6.6 ± 3.7	p<0.05***
Number of Refluxing Units (P/S) **	283 (226/57)	101 (74/27)	
Mean Injected Volume (mL)	0.63 ± 0.46	0.97 ± 0.47	p<0.05***
Success (at first injection)	90.5%	62.4%	p<0.05***

* **G/B =** Girls/Boys;

** **P/S =** Primary/Secondary;

*** Mann Whitney U

In PPC group, which documented a significantly higher success rate, we analyzed the relation between the success rate and the amount of injection material throughout the years of our practice. There was no statistically significant difference between the years regarding distribution of VUR grades, body weight, heights, and age at operation (p > 0.05, Kruskal Wallis test) (Table-2). The mean duration of follow-up was 37.9 ± 18.7 months. Overall reflux resolution rate with initial injection using PPC was 91.2% and increased to 92.4% after repeat injections. Reflux resolution rate and mean injected volumes through the years are summarized in Table-3.

**Table 2:** VUR grade distribution through the years in PPC group.

Years	VUR Grades (PPC Group)					
	1	2	3	4	5	Total
**2009**	1	5	3	11	1	21
**2010**	1	8	21	22	14	66
**2011**	6	8	24	14	16	68
**2012**	0	7	13	12	9	41
**2013**	2	9	16	9	9	45
**2014**	7	5	16	9	5	42
**Total**	**17**	**42**	**93**	**77**	**54**	**283**

**Table 3:** Reflux resolution rate and mean injected volumes through the years in PPC group.

Years	Reflux Resolution Rate	Mean Injected Volume
**2009**	90.5%	0.81
**2010**	93.9%	0.91
**2011**	95.6%	0.71
**2012**	80.5%	0.53
**2013**	91.1%	0.46
**2014**	90.5%	0.26

**Figure 1:**
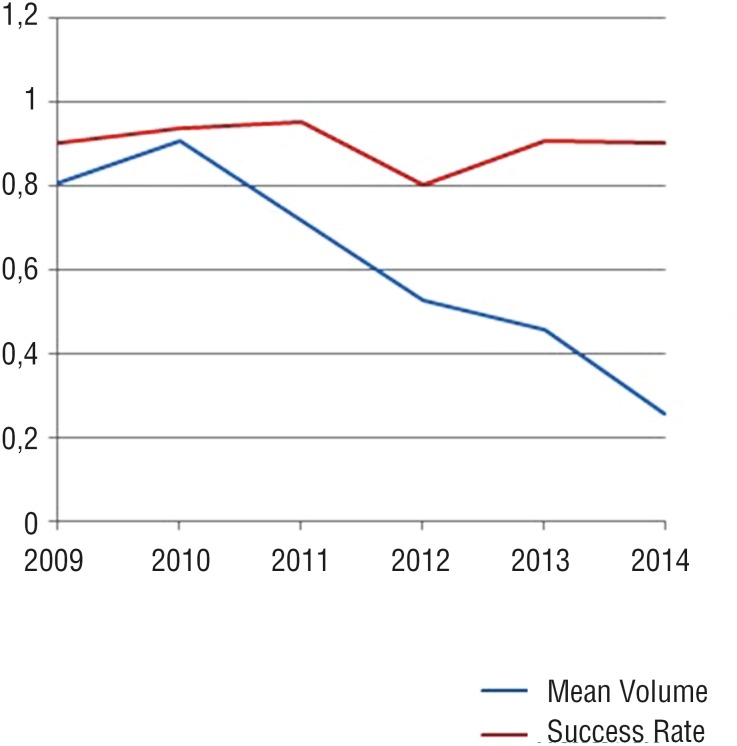
The success rate did not change significantly through the years.

Besides the significant difference between PPC and DxHA groups, mean injected material volume was 0.64 mL per ureter in PPC group; however, it gradually decreased in the study period. Mean injected volume per year decreased from 0.81 mL in the first year to 0.26 mL in the last year of the study period (67.8% reduction). In the meantime, the success rate did not change (Figure-1) (p > 0.05, chi - square).

Ureteral obstruction was noted in 8 of 283 injected ureters (2.8%) in 7 patients in PPC group. Obstructions were observed at 1 day to 11 months of time intervals after injection. Four of these patients were managed with temporary double - J stenting. Open ureteroneocystostomy was performed on the other three patients who did not benefit from temporary stenting. During the open surgery a fibrous capsule surrounding the substance and mild fibrosis was noted around the ureter, which did not complicate the ureteric dissection. When we retrospectively analyzed these cases, in two of them we found that there were beak sign in VCUG before the treatment that we did not appreciate it. We didn't encounter any obstruction cases in DxHA group so far.

## DISCUSSION

Starting with polytetrafluoroethylene (PTFE), many bulking agents with their own advantages and disadvantages have been used for endoscopic correction of VUR (8). Among them, dextranomer hyaluronic acid (Dx / HA) is the most widely used material (7, 9). Its biodegradable nature was suggested to induce minimal inflammation. Endoscopic treatment became the most popular model for VUR especially after its approval by the FDA in 2001. Overall success rate of the Dx / HA is reported between 68 – 92% (8). Polyacrilate Polyalcohol Copolymer is a relatively new material. Short and midterm results are encouraging to use it for the treatment of VUR (10, 11). One multicenter study reported its success rate as 93.8% after the first injection (10). Our success rate of 91.2% at first injection with PPC was also satisfactory, taking into account the high number of units with grade 5 VUR (19.1%) in our series.

After 20 years of experience with other materials, we switched to PPC as a bulking agent in 2009. A retrospective review of our experience with Dx / HA and PPC revealed increased success rate with less material using PPC. Some other studies reported similar results recently (12, 13). We documented a significant reduction (to almost one fourth of the initial volume used early in our experience) in injection volume within the last 5 years of the study period. Initial mean injection volume with PPC was similar to that of the previous agent Dx / HA. We realized in time that we were obtaining a so - called “volcano - type” mound with less material, but it took us some time to stop trying to reach the habitual injection volumes acquired through earlier experience with DxHA. As we got used to this new material's different characteristics, especially its extraordinary compressibility, we gradually decreased injection volume. We admit that we injected more than necessary at the beginning of our experience with this new material. The impression that the amount of material needed was decreasing over the years led us to evaluate our outcomes. Despite this significant change in injection volume, success rate did not decrease through the years with PPC. Two previously reported studies emphasize an opposite trend with Dx / HA (7, 14). Sorensen et al. pointed out a tendency of North American surgeons who used more vials of Dx / HA to achieve success. In their study, most patients were treated with a single vial and only 11% received 3 or more vials initially; however, over time, the number of patients receiving 2 vials significantly increased and the number of cases receiving 3 vials and more tripled (36%) (7). Lee et al. reported an increase in the injected volume in the second half of their experience with Dx / HA (15). Our contrast results with PPC are probably related to the molecular features of the material, such as particle size and compressibility. Particle diameter of PPC is more than 300 μm and with this size, it is larger than most other bulking agents (16).

Vesicoureteral obstruction is a rare but serious complication of endoscopic VUR treatment. Several studies examined the possible reasons of obstruction, mainly focusing on unnoticed refluxing obstructing megaureter, technical aspects, and type of injection material. Ureteral obstruction has been encountered in 7 patients so far after endoscopic treatment in our series. Intraoperative findings of ureteroneocystostomy were consistent with congenital refluxing obstructing megaureter in 2 of them. Aaronson et al. reported 2 cases with obstruction following subureteric injection with Dx / HA, and attributed obstruction to megaureter with the distal aperistaltic segment and cautioned against endoscopic treatment for these cases (17). Obstruction has also been related to the double hit technique with PPC (18). Three of our cases with obstruction were treated with this method. Different studies reported post - injection obstructions with almost all type of bulking agents mostly of unknown etiology (19-22). Our limited experience with obstructed ureters could not reveal a relation between obstruction and injected material volume. Some recent studies documented late obstructions with PPC and Dx / HA even after 5 years (19). Long term follow-up and randomized prospective studies are necessary in order to clarify this issue.

One of the weaknesses of our study is its retrospective nature and the absence of a control group. There is also no guideline or a study describing where one should stop injecting during the procedure. Instructions defining subureteric injection usually suggest the volcano - like appearance as a goal one must achieve during injection showing the pictures of it, which is not objective at all.

## CONCLUSIONS

Different bulking agents may require different injection volumes to achieve the same success rate in endoscopic treatment of vesicoureteral reflux. Polyacrilate polyalcohol copolymer is a new and effective bulking agent with different features, which ensures high success with less material. Habits gained with previous experience in terms of other materials should be revised while using a new agent.
